# Estimating soil organic carbon changes in managed temperate moist grasslands with RothC

**DOI:** 10.1371/journal.pone.0256219

**Published:** 2021-08-20

**Authors:** Asma Jebari, Jorge Álvaro-Fuentes, Guillermo Pardo, María Almagro, Agustin del Prado

**Affiliations:** 1 Basque Centre for Climate Change (BC3), Edificio Sede no. 1, Planta 1, Parque Científico de UPV/EHU, Barrio Sarriena s/n, Leioa, Bizkaia, Spain; 2 Estación Experimental de Aula Dei (EEAD), Spanish National Research Council (CSIC), Zaragoza, Spain; Universidade de Santiago de Compostela, SPAIN

## Abstract

Temperate grassland soils store significant amounts of carbon (C). Estimating how much livestock grazing and manuring can influence grassland soil organic carbon (SOC) is key to improve greenhouse gas grassland budgets. The Rothamsted Carbon (RothC) model, although originally developed and parameterized to model the turnover of organic C in arable topsoil, has been widely used, with varied success, to estimate SOC changes in grassland under different climates, soils, and management conditions. In this paper, we hypothesise that RothC-based SOC predictions in managed grasslands under temperate moist climatic conditions can be improved by incorporating small modifications to the model based on existing field data from diverse experimental locations in Europe. For this, we described and evaluated changes at the level of: (1) the soil water function of RothC, (2) entry pools accounting for the degradability of the exogenous organic matter (EOM) applied (e.g., ruminant excreta), (3) the month-on-month change in the quality of C inputs coming from plant residues (i.e above-, below-ground plant residue and rhizodeposits), and (4) the livestock trampling effect (i.e., poaching damage) as a common problem in areas with higher annual precipitation. In order to evaluate the potential utility of these changes, we performed a simple sensitivity analysis and tested the model predictions against averaged data from four grassland experiments in Europe. Our evaluation showed that the default model’s performance was 78% and whereas some of the modifications seemed to improve RothC SOC predictions (model performance of 95% and 86% for soil water function and plant residues, respectively), others did not lead to any/or almost any improvement (model performance of 80 and 46% for the change in the C input quality and livestock trampling, respectively). We concluded that, whereas adding more complexity to the RothC model by adding the livestock trampling would actually not improve the model, adding the modified soil water function and plant residue components, and at a lesser extent residues quality, could improve predictability of the RothC in managed grasslands under temperate moist climatic conditions.

## Introduction

Temperate grasslands, which cover 1.25 × 10^9^ ha globally, are important sinks of SOC, containing approximately 12% of the global SOC pool [1]. Changes in grassland management (e.g., stocking rate, fertilisation) are frequent in temperate conditions affecting SOC dynamics [2–4]. Grasslands ecosystems under temperate moist conditions are subject to processes that may differ from arable systems in regards with SOC sequestration. In particular, below-ground plant residues in grasslands provide important C inputs for soil C sequestration [5]: Grassland species allocate more C below-ground than cereals [6] and below-ground C has longer residence time than above-ground C [7]. Moreover, rhizodeposition is an important source of C inputs [8], which is rarely quantified and still remains the most uncertain component of soil C fluxes in terrestrial ecosystems [6].

Furthermore, grazing and wheeling by vehicles can cause damage soil and vegetation structure by trampling and poaching, both affecting plant production, and the potential amount of C inputs causing soil C loss [4,9]. Under temperate moist conditions, precipitations are high and winters are relatively mild with a relatively long growing season, susceptible to poaching [10]. Poaching is a common soil damage problem of livestock treading which has not been extensively simulated in grazing ecosystems [11]. Also, temperate moist climatic conditions imply that soils are wet-saturated during certain wet periods in which decomposition of organic matter is limited [12].

Therefore, improving the methods to estimate SOC stock changes in managed grasslands is key to obtain reliable estimates of SOC [13] and determine the real contribution of livestock to the net global greenhouse gas emissions.

Recent research in temperate grasslands has shown that grasslands can act either as C sink [14,15] or source [16] depending on how animals, vegetation, soil, climate, and management practices interact with each other [17–19]. To study the long-term responses of SOC changes in grasslands, we can use both data from long-term field trials [20,21] and simulation models. Models allows to obtain complementary information to trials, for example, hypothesis forming or/and to predict long-term responses of grasslands to climate change and management [22]. For strategic studies, e.g. assessing potential of grasslands to sequester SOC in a region, simple soil models, e.g. RothC [23], ICBM [24], C-Tool [25] and Yasso07 [26] are most useful as they require a limited and easily available input data. The RothC model, originally developed for arable soils, is one of the models that has been most widely validated and effectively used for different cropland and grassland systems at different spatial scales [27–29].

In general, RothC showed a good performance under grassland ecosystems [30,31]. Studies using RothC for grassland ecosystems have required specific initialization [32,33] using information from long term grassland experiments [34]. On the other hand, there are also several limitations to RothC particularly under managed moist grasslands. For instance, RothC presented a limitation considering management [35]. Despite the number of possible interactions in grassland systems, e.g. between plant, soil and animals, RothC simplified the effects of different management affecting some of these processes on grasslands and indirectly simulates grazing activity by altering the amount of total plant C inputs. As for animals C inputs, RothC offers default quality values for C inputs from grazing animals or manure applications. Moreover, the model does not consider the trampling effect on soil physical conditions related to grazing [29]. Besides, under temperate moist climatic conditions, RothC model is unable to adequately predict C dynamics in waterlogged soils [36], which imply oxygen limitation and thus a decline in decomposition rate [12]. Furthermore, as a general limitation, regarding plant residues, RothC does not differentiate between above- and below-ground C inputs [33].

Considering the model limitations, we aimed to introduce modifications to RothC and assess the ability of the proposed modifications to predict the measured SOC stocks from intensive grassland sites under moist climatic conditions. To adapt RothC to temperate moist managed grassland, we hypothesized that the aforementioned factors (i) could be easily implemented in RothC, (ii) significantly affect SOC changes and (iii) could improve RothC predictions of SOC changes. To evaluate the suggested modifications, we assessed the model performance against published experiments through a stepwise approach, as well as its sensitivity to the main modifications.

## Materials and methods

### RothC model overview

The RothC -26.3 [23] model divides the SOC into five fractions, four of them are active and one is inert (i.e., inert organic matter, IOM). The active pools are: decomposable plant material (DPM), resistant plant material (RPM), microbial biomass (BIO) and humified organic matter (HUM). The decomposition of each pool (except IOM) is governed by first-order kinetics, characterized by its own turnover rate constant and modified by environmental factors related to air temperature, soil moisture and vegetation cover, which are the main input parameters to run the model. Incoming plant C is split between DPM and RPM, depending on the DPM: RPM ratio of the particular incoming plant material or organic residue. Both of them decompose to produce BIO, HUM and evolved CO_2_. The proportion that goes to CO_2_ and to BIO + HUM is determined by the clay content of the soil which is another input to the model.

The model uses a monthly time step to calculate total SOC and its different pools changes on years to centuries time scale.

### RothC tested modifications

Four modifications were proposed and tested in this study to the RothC excel version (*“Rothc_single_layer_4_active_pools_Feb_2013”*): (i) extensions of soil water content function up to saturation; (ii) enlargement of C input pools to account for the diversity of applied exogenous organic matter (EOM) from ruminant excreta; (iii) affinition of plant residue components and quality variability; and (iv) the trampling/poaching effect of grazing animals.

#### Soil water saturation in RothC

RothC contains a minimum rate modifying factor of 0.2, when soil moisture is at minimum moisture capacity (i.e., at the extreme of water limitation). However, no correction is applied under water saturation and when soil is oxygen limited. In order to represent the reduction in the decomposition rate above field capacity [37–39], the rate modifying factor for moisture was assumed to follow a linear decline trend until a minimum rate of 0.2 (20%), at saturation conditions, as suggested by [40] in the ECOSSE model.

ECOSSE soil moisture function was derived from SUNDIAL and RothC models under anaerobic and aerobic conditions and based on Rothamsted field experiment [41]. The model was designed for use across a range of land uses, and water contents are included up to saturation [40]. It was evaluated under European conditions and showed a good performance [42].

Soil water contents at saturation and field capacity conditions are estimated by considering soil properties related to soil texture as described by [43].

The conversion from soil water content to soil moisture deficit (SMD_i_, mm) used in RothC referred to [44] (S1 Appendix in S1 File).

#### Exogenous organic matter diversity (EOM)

Exogenous organic matter partition for the RothC model was estimated by [45], based on an indicator of potential residual organic C in soils (IROC), which is derived from Van Soest fractions and the proportion of EOM mineralized during 3 days of incubation. Similarly [46], improved the prediction of SOC stocks in amended soils by fitting the RothC partitioning pools of different EOM to the respiratory curves. Such adjustment of the partition of EOM into RPM, DPM and HUM entry pools of RothC provided a successful fit and had been reproduced in other studies [47]. However, the above-mentioned studies have summed up all the different animal excreta into one category and did not distinguish excretions from different animal types (e.g., ruminants, pigs…). In order to capture the specific characteristics of ruminant excreta, we developed a methodology based on [48] as illustrated in S1 Fig in S1 File. In this study [48] proposed a partition of the C inputs from excreta into RothC pools based on the relationship between lignin content (Van Soest fractions) and anaerobic biodegradability, estimated as follows (Eq (1)):

B=0.905×exp(−0.055×lig(%)) (1)

Where B is biodegradability and Lig is lignin content as % of Volatile Solids (VS).

The Van Soest fractions are then partitioned into the pools of RothC based on its degradability, represented by the parameter B (i.e, lignin, holocellulose and solubles). A fraction of lignin is allocated into the HUM pool, representing the most resistant material. The rest of the lignin and the most resistant fraction of holocellulose and solubles are assigned to the RPM, while the most labile fraction of holocellulose and solubles are allocated to DPM.

This is expressed as VS %, following the equations.


HUM=Lig×(1−B) (2)

RPM=lig×B+(Holocellulose+Solubles)×(1−B) (3)

DPM=(Holocellulose+Solubles)×B (4)

The Van Soest fractions were derived from literature review for animal excreta of ruminants. As a result of this review, we identified large variability in animal excreta’s fractions (lignin: S2 Fig in S1 File and soluble: S3 Fig in S1 File). These differences were associated to a diverse array of factors and especially those in relation with the animal diet composition (e.g., high concentrate diet generally would imply lower lignin content in the ruminant´s excreta). For the ruminant excreta quality to the RothC entry pools, we used as input to the above questions an average value for the different fractions considered (data from S2 and S3 Figs in S1 File) (Table 1). Additionally, in a separate exercise, we evaluated how the impact of uncertainties of these fraction values could lead to uncertainties on the SOC results. For this exercise, both extreme values (i.e., maximum and minimum) were assessed using a sensitivity analysis (See Sensitivity analysis section).

**Table 1:** Ruminant excreta quality and its fitting to the RothC entry pools (based on scientific literature review).

	RothC Pools		
	HUM	RPM	DPM
**Ruminant excreta**	0.1	0.6	0.3

#### Plant residue: Components and quality

The RothC model does not distinguish between above- and below-ground plant residues. We hypothesise that accounting for month-to-month changes in plant residue quality may improve RothC predictions under wet conditions, while not adding too much complexity to the modelling approach. Regarding plant C inputs distribution, RothC is known to be relatively insensitive to the distribution of C inputs through the year [49].

Model users generally use above-ground residues as surrogate for total plant C inputs and do account less for root inputs in RothC [33]. Here we separated the plant residue C inputs into three components (i.e., above-ground residues, below-ground residues and rhizodeposits). The structure of C input derived from plant residues in RothC modified model is as illustrated in S4 Fig in S1 File. Parting from above-ground biomass, we used root to shoot (R:S) ratio to estimate below-ground biomass (when its value is not available). We assumed N fertilisation as the main driver for R:S ratio in grasslands as many studies have proved the strong dependence of the latter on N inputs [5,50]. We referred therefore to [50] equation for RothC C input parameterisation under temperate grasslands in order to consider the fertilisation effect on the R:S ratio:

$$R:S=4.7375e^{-0.0043.\;N\;input}$$ (5)

Where R:S is the Root: Shoot ratio and N input is nitrogen fertilisation expressed in kg N ha^-1^ year^-1^.

Unlike in annual croplands, in perennial grassland ecosystems, below-ground C biomass does not correspond to the below-ground residue. Instead, below-ground residues correspond to 50% of the total below-ground C biomass [50] since the average annual root turnover of grasslands has been estimated to be 50% in the temperate zone [51].

Regarding rhizodeposition estimation, we referred to an extensive literature review in which net rhizodeposition-to-root-ratio from grasslands was estimated to be 0.5 [6].

We assumed a C concentration of 45% of the plant biomass [52].

Plant residue quality (biochemical composition), as one of the main drivers of decomposition, is represented in the RothC model by the DPM:RPM ratio (i.e., ratio of rapidly and slowly decomposing pools), which can be obtained by optimization to obtain the best fit according to different land use types. For instance, for most agricultural crops and improved grasslands, RothC uses a DPM: RPM ratio of 1.44 (i.e. 59% of the plant material as DPM and 41% as RPM). For unimproved grasslands and scrubs (including Savannas) a ratio of 0.67 is used [23]. Plant residue quality is variable in time and depends on several factors (e.g., maturity stage, climate variables and nitrogen fertilisation) [53,54].

In order to fit the DPM: RPM ratio to the specific conditions of temperate grasslands, including its variability over the year, we used the stepwise chemical digestion (SCD) method [55], already used by [56,57]. For simplicity, we assumed that the DPM pool could be approximated to the C in the Neutral Detergent Soluble (NDS), and the RPM pool as the C in the Neutral Detergent Fiber (NDF) (i.e., holocellulose and lignin fractions).

Regarding below-ground plant material quality, the quantity of lignin itself is the main potential driver of differential degradation between both above- and below-ground plant components [58]. Therefore, we added up the difference of lignin percentage of ~ 8% (between above- and below-ground parts) to get the below-ground RPM pool, referring to [59].

The DPM pool is then derived by subtraction according to the equation:

DPM(%)=100−RPM(%) (6)

Finally, we assumed that the C inputs derived from rhizodeposition are transferred to DPM of the RothC because of the expected rapid decomposition of this labile substance by rhizosphere microorganisms [60].

#### Animal trampling effect: Poaching

We hypothesise that accounting for animal trampling may improve RothC predictions, while not adding too much complexity to the modelling approach. The trampling effect generally depend on stocking density, soil moisture content, soil texture, and the presence/absence of a protective vegetation cover [61]. Apart from the stocking rate, the remaining factors were reflected in the RothC default model. In this context, we developed a simple poaching modification based on available data obtained from temperate moist grassland studies [62–64], considering that our modelling should be validated apart. The main objective of introducing the poaching effect was to predict the level of soil damage and its impact on plant C inputs as a function of soil moisture, soil compaction and degradation under grazing conditions (e.g. under different stocking rates) (S5 Fig in S1 File). Soil moisture is estimated in RothC using the Soil Moisture Deficit (SMD) value that considers rainwater inputs and soil texture properties (i.e., clay content). According to [65,66], we used SMD as a proxy for soil moisture to predict when soil water conditions are likely to lead to hoof damage. For simplification reasons, we assumed water saturation conditions from SMD = -10 mm onwards (according to the soil moisture modification), as a condition of poaching occurrence as in [64,67]. Livestock density has an effect on the level and extent of treading damage [64,68] illustrated by hoof print depth and soil surface deformation [62]. Depending on the soil surface deformation of a treading event, the pasture production is reduced [63] and thus its plant C input [4] (S4 and S5 Figs in S1 File). The main equations related to the conceptual diagram of poaching modification are described in S1 Appendix in S1 File.

As the poaching effect in temperate grazing systems seems to cause only short-term reduction in pasture plant production but there is a relatively fast recovery to these damages [62,69], we considered that plant C input reduction due to poaching effects would only occur in months when soil was prone to poaching.

### Study sites and input datasets

#### Study sites description

In order to validate the proposed modifications, we used data from four studies of European managed grasslands having temperate conditions and being characterized by precipitations > 1000 mm, and grass and clover mixture. The grassland sites (Laqueuille intensive grazing grassland, Oensingen intensive cutting grassland, Easter Bush intensive grazing grassland and Solohead dairy research farm) (Table 2) were mainly selected from the FLUXNET program (http://www.fluxnet.ornl.gov/; [70]. Information on geographic and climatic characteristics, soil properties and management of the different sites are listed in the Table 2. More details are provided in S2 Appendix in S1 File.

**Table 2:** Location, climate, soil properties, management type and input data to the model of the grassland study sites (available through the European Fluxes Database Cluster: http://www.europe-fluxdata.eu (except Solohead farm).

Site name and references	Laqueuille [71]	Oensingen [72]	Easter Bush [73,74]	Solohead farm [75]
**Country**	France	Switzerland	United Kingdom	Ireland
**Altitude (m)**	1040	450	190	150
**Latitude**	45^o^ 38´N	47^o^ 17´N	55^o^ 52´N	52°51´N
**Longitude**	02^o^ 44´E	07^o^ 44´E	03^o^ 02´W	08°21´W
**Mean air temperature (** ^**O**^ **C)**	7	9	9	10.6
**Mean annual precipitation (mm)**	1012	1263	1031	1017
**Simulation period**	2004–2012	2004–2011	2004–2011	2004–2011
**Grassland type**	Intensive semi-natural permanent grassland	Intensive permanent grassland	Intensive permanent grassland	Intensive permanent grassland
**Management (Mowing/Grazing frequency)**	• Grazing by heifers (May to October)	• Grass mowing (4 times a year) • No grazing	• Grazing all year round by cattle and sheep	• Grazing by dairy cows February to November • Mowing
**Annual production (t ha** ^**-1**^ **yr** ^**-1**^ **)**	7.1	7.5	5.6	13.5–14.7
**Stocking rate (LSU ha** ^**-1**^ **yr** ^**-1**^ **)**	~1	-	0.83	~2
**Total N fertilisation**	Mineral fertilisation in three splits: 210 kg N ha^-1^ yr^-1^	Solid ammonium nitrate or cattle slurry at the beginning of each growing cycle (after the previous cut): 214 kg N ha^-1^ yr^-1^	Ammonium nitrate fertiliser was applied to the field 3–4 times per year, usually between March and July (~ 229 kg N ha^-1^ yr^-1)^	N fertiliser ~183 kg N ha^-1^ yr^-1^
**SOC in the topsoil (Mg C ha** ^**-1**^ **yr** ^**-1**^ **)**	114 ± 1.48 (20 cm depth) in 2004 125.8 (20 cm depth) in 2008 121±2.35 (20 cm depth) in 2012	64.7 (20 cm depth) in 2004 68.3±1.6 (20 cm depth) in 2011	93.26 (30 cm depth) in 2004 87.87 (30 cm depth) in 2011	137±6.5 (30 cm depth) in 2004 142.8±7.14 (30 cm deth) in 2008 148.8±7.16 (30 cm deth) in 2009 149.2±9.7 (30 cm depth) in 2011

**Soil properties**	The soil is an Andosol (20% clay, 53% silt and 27% sand) with 11% carbon and 18% organic matter.	The soil is classified as Eutri-Stagnic Cambisol (FAO, ISRIC and ISSS, 1998) developed on clayey alluvial deposits. Clay contents between 42% and 44% induce a total pore volume of 55% and a fine pore volume of 32% (permanent wilting point)	The soil type is an imperfectly drained Macmerry soil series, Rowanhill soil association (Eutric Cambisol) with a pH of 5.1 (in _H2O_) and a clay fraction of 20–26%.	The predominant soils are poorly drained gleys (90%) and grey-brown podzolics (10%) with a clay loam texture and low permeability (28% clay, 35%silt)
**Grass type**	Grass clover mixtureThe dominant grass are *Dactylis glomerata*, *Trisetum flavescens*, *Poa pratensis and Agrostis capillaris*	Grass clover mixture	>99% rye grass (*Lolium Perenne*) and < 0.5% white clover (*Trifolium repens*)	rye grass and white clover (20 to 25%)
**Estimated/Measured R:S ratio**	1.92 (Estimated)	1.46 (Measured)	1.77 (Estimated)	0.88 (Measured)
**Measured Above-ground C (t C ha** ^**-1**^ **)**	0.89	1.3	0.5	4.8
**Estimated/Measured Below-ground C (t C ha** ^**-1**^ **)**	1.71 (Estimated)	1.9 (Measured)	0.88 (Estimated)	4.2 (Measured)
**Plant residue components (t C ha** ^**-1**^ **)**	C_a_ = 0.19; C_b_ = 0.86; C_r_ = 0.86	C_a_ = 0.2; C_b_ = 0.95; C_r_ = 0.95	C_a_ = 0.1; C_b_ = 0.44; C_r_ = 0.44	C_a_ = 0.9; C_b_ = 2.1; C_r_ = 2.1
**Biomass quality**	NDF_a_ ranges from 0.55 to 0.67 NDF_b_ ranges from 0.63 to 0.75 NDF_r_ = 0	NDF_a_ ranges from 0.56 to 0.68 NDF_b_ ranges from 0.64 to 0.76 NDF_r_ = 0	NDF_a_ ranges from 0.55 to 0.69 NDF_b_ ranges from 0.63 to 0. 77 NDF_r_ = 0	NDF_a_ ranges from 0.51 to 0.64 NDF_b_ ranges from 0.59 to 0.72 NDF_r_ = 0
**C input derived from ruminant excreta (t C ha** ^**-1**^ **yr** ^**-1**^ **)**	0.54	0.47	0.75	2.3

NDF_a_, Neutral Detergent Fiber corresponding to resistant above-ground plant material; NDF_b_, Neutral Detergent Fiber corresponding to resistant below-ground plant material; NDF_r_, Neutral Detergent Fiber corresponding to rhizodeposits.

C_a_, above-ground plant C input; C_b_, below-ground plant C input; C_r_, plant C input corresponding to rhizodeposition.

#### Input data for the model and main assumptions

Plant carbon inputs in the different sites were estimated depending on the available data using the method described in the section “Plant residues: Components and quality”. For the Laqueuille site, average above-ground C residue was obtained from available measured data and it represented 20% of above-ground C standing biomass (Table 2). We used the R:S ratio to estimate below-ground biomass from average measured above-ground standing biomass. Below-ground C residues were assumed to be 50% of the below-ground C biomass [50] (Table 2). For the Oensingen site, average above- and below-ground C biomass were obtained from [72]. We used the same assumption as [50] for cutting grasslands, assuming that 30% of the above-ground biomass is not harvested, and that only 50% of that fraction is turned over annually and becomes available for soil organic matter formation [76] (Table 2). To estimate below-ground C residue, we used the same assumption as commented for Laqueuille site (Table 2). The same assumptions were considered for the grazing Easter Bush site. From the average measured above-ground biomass we assumed only 20% as residues as in the Laqueuille grazing site and the same hypothesis for the below-ground C residue as in the other previous sites (Table 2). For Solohead dairy research farm, we used as input the available measured data of above- and below-ground C biomass and used the same assumption for above- and below-ground C residues as all the previous sites (Table 2). Finally, for the rhizodeposition as commented previously, we used an annual net rhizodeposition-to-root ratio of 0.5.

The proportions of plant C input added to the soil in each month were set as the pattern of inputs typical of European grasslands suggested by [49]. Referring to plant residue quality we ascribed RPM and DPM pools related to NDF and NDS, respectively for each plant residue component (as described in the sub-section “Plant residues: components and quality”).

The C animal excreta in Laqueuille grazing grassland were derived from [77] referring to the C intake grass-based rations, as the management is a continuous grazing from May to end of October without additional feed supply [71]. Therefore, we estimated the C animal excreta as 32% of the measured C intake using average values for the simulation period 2004–2012 [77]. Annual C derived from cattle slurry in Oensingen site were estimated from [78] as an average of the provided years. Carbon input from grazing animal excreta was estimated the same as in Laqueuille site, while annual C input derived from organic fertilisation for Easter Bush was deduced from [73] during the period 2004–2010 as 0.32 Mg C ha^-1^yr^-1^. The same method was used to estimate annual total N fertilisation and annual stocking rate of this site. For Solohead dairy research farm, C input derived from animal excreta were calculated the same as in Laqueuille site and all other input data were estimated as average annual from the same study [75].

The different input data to the model regarding management, soil properties to estimate soil water content at saturation and field capacity conditions [43], as well as grass type to characterise the plant residue quality for the different study sites are illustrated in Table 2.

#### Model initialisation and running

For RothC initialisation, since radiocarbon measurements are costly and rarely performed routinely, we used the pedotransfer functions established by [79] to estimate all active C pools from initial provided measured SOC stocks. The initial IOM pool, according to these pedotransfer functions was set to match the equation proposed by [80]:

$$IOM=0.049\;{TOC}^{1.139}$$ (7)

We modelled SOC dynamics from the different study sites using a stepwise approach. First, we used the default RothC version (RothC_0) and, subsequently we progressively added the different modifications tested (Table 3): (i) ruminants excreta (RothC_1 modification); (ii) plant residue components and its characteristics (RothC_2 modification); (iii) saturation conditions for soil water function (RothC_3 modification) and (iv) soil poaching (RothC_4 modification).

**Table 3:** RothC versions tested in the study with the modification included in each version.

RothC version	Modifications
**RothC_0**	RothC default version
**RothC_1**	RothC_0 + ruminant excreta characteristics
**RothC_2**	RothC_1 + plant residue characteristics and its variability
**RothC_3**	RothC_2 + saturation conditions for soil water function
**RothC_4**	RothC with all modifications: RothC_3 + inclusion of poaching effect

Soil organic carbon stocks were simulated at 20 cm depth for Laqueuille and Oensingen and at 30 cm depth at Easter Bush and Solohead dairy farm.

### Model evaluation

We used different performance indices and threshold criteria based on [81] (S1 Table in S1 File). The ability of each modification to improve SOC dynamics simulation was evaluated using the root mean square error (RMSE), mean difference of simulations and observations (BIAS) and the model efficiency (EF) (S1 Table in S1 File).

#### Sensitivity analysis

Several studies have indicated that the RothC model is most sensitive to C inputs [82–84]. In our study, analyses were performed to test the sensitivity effect on SOC changes of the different modifications (other than C inputs) implemented in the model, using RothC_4. Model sensitivity was expressed as an index, which considered different input values related to the modifications (i.e., plant residues quality, ruminant excreta quality and soil moisture up to saturation) from minimum to maximum (Table 4) and then the output values were analysed according to the following index [81].


$$Sensitivity\;index=\frac{max(Pi)-min(Pi)}{max(Pi)}$$ (8)

**Table 4:** Model modified components used for the sensitivity analysis and their interval values.

Modified component	Proxy	Interval for possible values
**Plant residues quality (e.g., perennial grass)**	NDF	30–70%
**Quality of ruminant excreta (e.g., cattle slurry)**	Lignin	9–28%
**Soil moisture up to saturation**	Rate modifying factor for moisture	0.2–1

Where max (Pi) is the maximum output value and min (Pi) is the minimum output value.

We used NDF as a proxy for RPM in relation with plant residues quality (Table 4), assuming that NDF varies from 30 to 70% as minimum and maximum values based on 15 papers (S2 Table in S1 File). We used the lignin fractions (% VS) as a proxy for EOM in relation with ruminant excreta quality assuming minimum and maximum values from literature values shown in Table 4. Similarly, for soil moisture variation, we tested minimum (0.2) and maximum values (1) of the rate modifying factor for moisture (Table 4).

## Results and discussion

### Measured versus simulated SOC stocks

**Fig 1:**
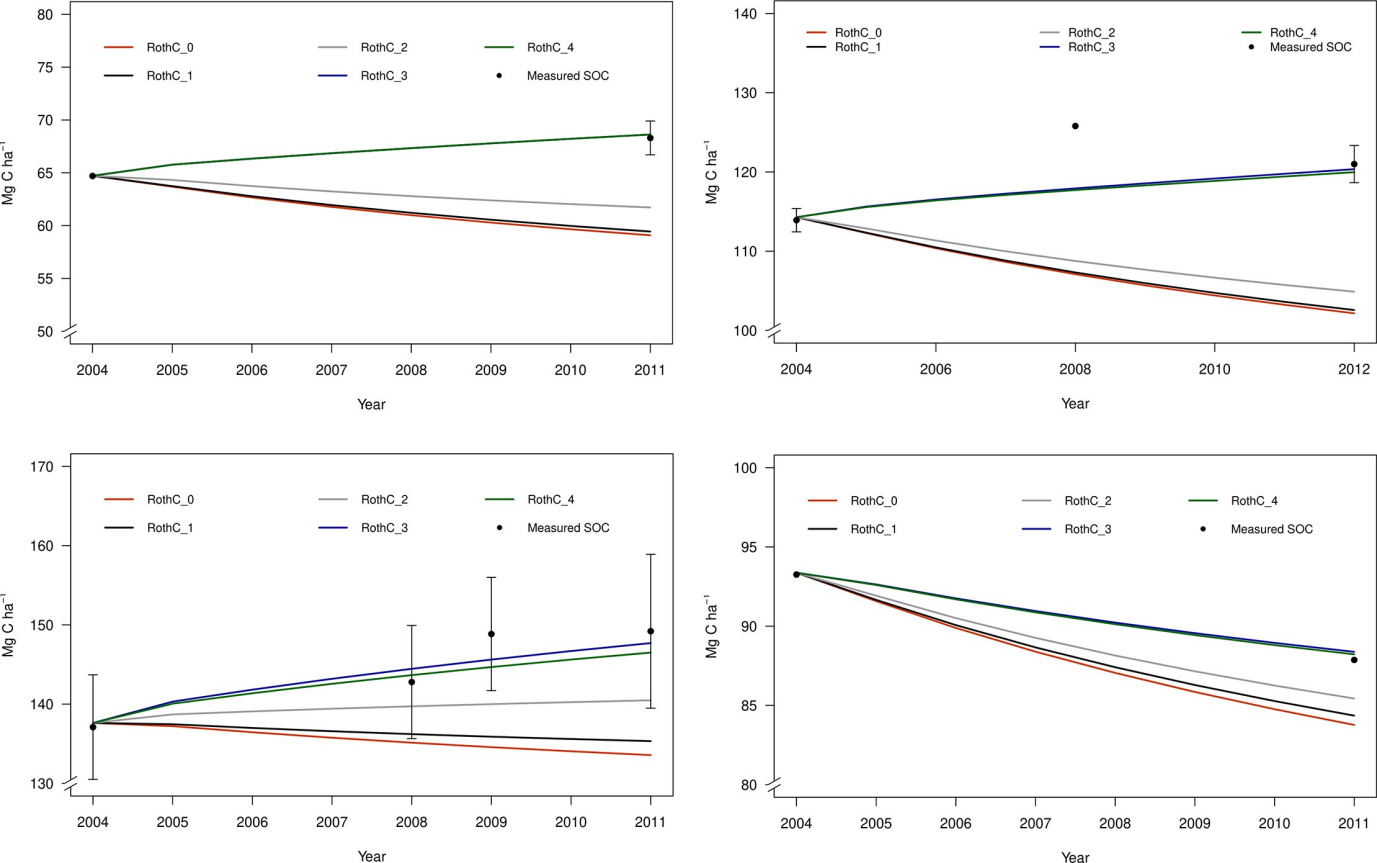
Measured and simulated annual SOC stocks (Mg C ha^-1^) using the default RothC model (RothC_0) and the modified RothC versions (RothC_1; RothC_2; RothC_3; and RothC_4) for the different validation sites. (a) Laqueuille intensive grazing grassland; (b) Oensingen intensive cutting grassland; (c) Easter Bush intensive grazing grassland; and (d) Solohead dairy research farm.

All four sites showed, in general, a similar pattern of annual SOC stocks with the RothC default version (i.e., RothC_0) as well as with the four modified versions (Fig 1). In all four sites, the lowest simulated SOC stocks were observed in the default model version (RothC_0). RothC_0, for Laqueuille, Oensingen and Solohead sites, simulated that SOC was reduced during the time of the experiment (Fig 1A, 1B and 1D), which was the opposite trend that measurements showed. For example, in the Laqueuille intensive grassland, SOC stocks predicted by the RothC_0 version decreased from 114 to 102 Mg C ha^-1^ whereas measured values increased from 114 to 121 Mg C ha^-1^ (Fig 1A).

By implementing changes to account for ruminant excreta quality (RothC_1) on the study sites, the model resulted in a slight increase in SOC in time. Moreover, this SOC increase was lower than that simulated by RothC_2 (Fig 1). Changes in the modification of plant residues (RothC_2) resulted in greater SOC increased values in time when compared with the previous modification (RothC_1) (Fig 1). The lower effect of the simulation of animal excreta characteristics in RothC_1 could be explained by the higher quantity of plant residues while adding the rhizodeposition component together with above- and below-ground components in RothC_2.

By introducing the soil moisture modification in RothC (RothC_3), the model simulated an increase in SOC stocks which, trend-wise, differs from the RothC_0 model, but coincides with measured data (Fig 1). For example, SOC stocks at the end of the simulation period in 2011 reached 88.38 Mg C ha^-1^ (RothC_3) compared to 83.7 Mg C ha^-1^ (RothC_0) in the Easter Bush intensive grazing grassland (Fig 1C). Soil moisture modification at saturation reduces decomposition rates values for very wet soils conditions. In fact, the 4 sites used in our study have soil water saturation during many months of the year (with an average of 8 months).

Including the poaching effect (RothC_4), resulted in slightly reduced SOC stocks compared with RothC_3, specially for the Solohead site (Fig 1A, 1C and 1D). This reduction in SOC stocks in RothC_4 compared with the RothC_3 version could be explained by the reduction in plant C inputs due to poaching that typically occurs at saturation conditions [4,85].

In general, the highest predicted SOC stocks values and the closest to the measured values at the end of the simulation period resulted after RothC_3 and RothC_4 simulations (Fig 1). For Laqueuille grassland intensive site, RothC_3 and RothC_4 were able to match the general trend of SOC increase (between 2004 and 2012) and the SOC stocks at the end of the simulation period, but not the change of SOC stocks corresponding to the year 2008. However, SOC simulation for Solohead research farm, using RothC_3 and RothC_4 modified versions were within the range of measured data of SOC stocks (Fig 1).

### Model performance

**Fig 2:**
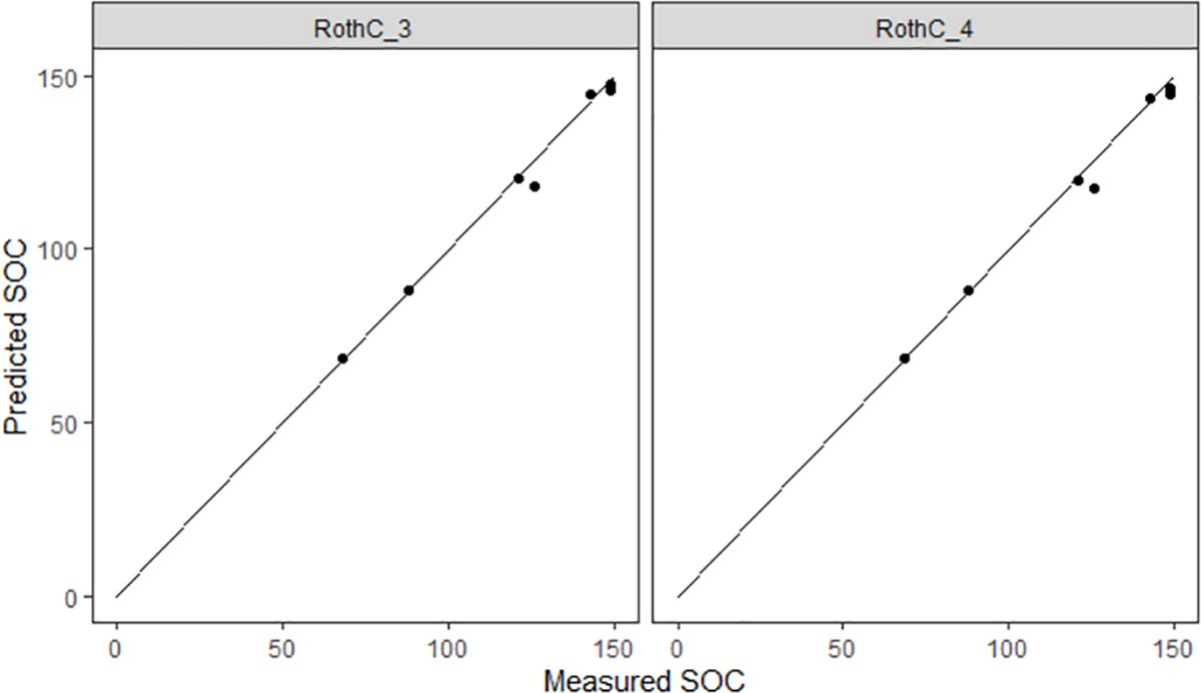
Measured versus predicted values of SOC stocks at the end of the simulation period using RothC_3 and RothC_4 model versions for the different study sites.

In general, the RothC default version (RothC_0) showed a good performance with an EF value of 78% (Table 5). However, the different cumulated modifications presented enhanced the predicting performance of RothC for these specific sites. In particular, simulated SOC stocks using the RothC_3 and RothC_4 versions almost matched measured values (Fig 2) achieving model efficiencies of 99% and 98% (Table 5). Therefore, these two modifications accurately predicted SOC changes.

**Table 5:** Root mean square error (RMSE) and mean difference of simulations and observations (BIAS) of SOC stocks (Mg C ha^-1^) for each model version and grassland intensive site and model efficiency (EF) and RMSE across sites.

Site	Performance test	RothC_0	RothC_1	RothC_2	RothC_3	RothC_4
**Laqueuille**	**BIAS**	**-18.77**	**-18.45**	**-16.56**	**-4.26**	**-4.55**
	**RMSE**	**15.21**	**14.95**	**13.43**	**4.53**	**4.67**
**Oensingen**	**BIAS**	**-9.22**	**-8.86**	**-6.58**	**0.32**	**0.32**
	**RMSE**	**13.49**	**12.97**	**9.64**	**0.47**	**0.47**
**Easter Bush**	**BIAS**	**-4.10**	**-3.52**	**-2.43**	**0.51**	**0.35**
	**RMSE**	**4.67**	**4.00**	**2.77**	**0.58**	**0.40**
**Solohead**	**BIAS**	**-12.52**	**-11.13**	**-6.88**	**-1.02**	**-2.00**
	**RMSE**	**8.85**	**7.89**	**5.03**	**1.55**	**1.98**
**All sites**	**RMSE**	**11.36**	**10.75**	**8.66**	**2.77**	**3.01**
	**EF**	**0.78**	**0.80**	**0.87**	**0.99**	**0.98**

The negative bias (reaching -18.8 in Laqueuille site) and the higher RMSE values obtained in RothC_0 compared with the different RothC modified versions indicated that the default RothC version underestimated SOC stocks, especially in the Laqueuille and Solohead sites, which presented the highest SOC content (Table 5). This confirmed the fact that the RothC model is unable to adequately predict soil C dynamics in organic or waterlogged soils [36]. In this context, adding the modification of the soil moisture function in RothC_3 reduced the bias and the RMSE (Table 5) and improved the general trend of SOC stocks compared with the default version RothC_0 in all simulated sites (Fig 1). RothC_0 assumes high decomposition rates with high soil moisture, but it does not consider the cessation of the decomposition process which occurs in high wet soils close to saturation conditions [86], frequent in temperate moist grasslands. The inclusion of the ruminant excreta quality in the model only slightly improved the SOC predictions in RothC_1 compared to RothC_0 (Table 5). In this context, [46,87] emphasised the importance of modifying the quality of residues to improve the model performance, concluding that the adjustment of DPM:RPM ratio led to better model performance than the use of default DPM: RPM values provided by the model.

Comparing RothC_1 and RothC_2 versions, it could be deduced that integrating quantity and quality distinction of plant residue in RothC_2, as a primary source of SOC [88], improved SOC predictions. Adding the modification of plant residues in terms of quantity and quality contributed to improve SOC simulation compared to the modification of specifying animal excreta quality. The improvement showed by plant residues modification, particularly in Solohead Research farm, could be explained by the higher sensitivity of the model to C inputs quantity compared to C inputs quality and the importance of including rhizodeposition together with above- and below-ground components in plant C input quantification. Indeed, as a fundamental source of C inputs, rhizodepostion was recommended to be added to the different plant residue components in SOC models [60,89], particularly RothC [90].

The poaching effect is assumed to reduce plant productivity and the potential amount of C inputs to the soil [4] and thus causing SOC loss [9]. Consequently, the poaching modification included in the RothC_4 version predicted reductions in SOC stocks compared to the RothC_3 version (Fig 1A, 1C and 1D). The reduction in SOC stocks is explained by the lower C inputs during the months when grazing occurs under saturation conditions. Only in the case of the Easter Bush site, the poaching modification contributed to improve SOC predictions in the RothC_4 version (Table 5, Fig 1C). A possible explanation to this improvement in the SOC predictions is that the soil in Easter Bush site is poorly drained and grazing by ruminants occurs all year round and thereby highly susceptible to poaching. In the same context [91], enhanced the original PASIM grassland constructing a simple and empirical model of the detrimental impact on vegetation of trampling by grazing animals by removing at each time step a fixed proportion of the above- ground biomass. However, it is important to point out the complexity of the poaching effect, as it induces more impacts other than the detrimental vegetation impact which are beyond the scope of our study. In this context [92], pointed out the inconsistency and limitation of the studies dealing with the grazing effect on SOC. Therefore, more robust experiments are needed in order to define the severity of the poaching effect according to soil moisture saturation, livestock density and soil type.

Therefore, particularly, RothC_3 showed the best agreement (Table 5, Fig 2), as the effect of the poaching modification added in RothC_4 is minimal and uncertain. In this sense, the poaching modification could be of major importance under heavy stocking rates or overgrazing management associated to SOC loss [93].

Testing the model performance based on each of the individual modifications for the different sites allowed improving our understanding of its impact to the model (Table 6). Soil moisture up to saturation conditions in the soil water function of RothC showed the best performance compared with the other modifications (Table 6). The modification of RothC water function at saturation conditions fit to the temperate moist climatic conditions, since the different study sites showed saturation conditions most of the year. However, the poaching effect alone contributed to reduce SOC stocks and thus the model performance, since the poaching effect is related to water saturation conditions [64]. The enhancement in the model performance showed by the quality of ruminant excreta depends on its quantity. Indeed, the BIAS reduction with ruminant excreta quality modification compared with the default version (Tables 5 and 6) was more important in the grassland sites with major ruminant excreta application (e.g., Solohead research farm). However, the plant residue modification showed a higher improvement compared with the ruminant excreta quality as it implies an increase in C inputs with the inclusion of the rhizodeposition component.

**Table 6:** Root mean square error (RMSE) and mean difference of simulations and observations (BIAS) of SOC stocks (Mg C ha^-1^) for each specific modification (i.e., soil moisture up to saturation, ruminant excreta quality, plant residue, poaching effect) to the model and grassland intensive site and model efficiency (EF) and RMSE across sites.

Site	Performance	Soil moisture up to saturation	Ruminant excreta quality	Plant residue	Poaching
**Laqueuille**	**BIAS**	**-7.27**	**-18.45**	**-16.88**	**-18.91**
	**RMSE**	**6.31**	**14.95**	**13.69**	**15.32**
**Oensingen**	**BIAS**	**-2.95**	**-8.86**	**-6.94**	**-**
	**RMSE**	**4.32**	**12.97**	**10.16**	**-**
**Easter Bush**	**BIAS**	**-1.29**	**-3.52**	**-3.02**	**-4.21**
	**RMSE**	**1.47**	**4.00**	**3.44**	**4.79**
**Solohead**	**BIAS**	**-7.17**	**-11.13**	**-8.27**	**-13.21**
	**RMSE**	**5.23**	**7.89**	**5.97**	**9.31**
**All sites**	**RMSE**	**5.51**	**10.75**	**9.19**	**12.19**
	**EF**	**0.95**	**0.80**	**0.86**	**0.46**

However, testing the model based on the combined effect of soil moisture up to saturation and poaching effect showed a great performance compared with the effect of excreta and plant residues for the different sites with a RMSE of 5.96 compared with 8.66 (Table 7). The modifications of soil moisture up to saturation and poaching effect reduced the BIAS compared with animal excreta and plant residue modifications for the different study sites, except for the Solohead research farm. This could be explained by the fact that the latter received higher C inputs derived from animal excreta and plant residues and lower water saturation conditions compared with the other sites (Table 2). The modifications of soil moisture up to saturation and plant residues presented the best performance among all sites (Table 7). Particularly, the plant residues modification implied an accounting for rhizodeposition component and thus a significant increase in C inputs compared with the minimum proportion of plant residues reduction induced by the poaching effect of grazing animals at saturation conditions. Therefore, the model modification with the greatest positive impact was soil moisture up to saturation (Tables 6 and 7). However, the impact of plant residues and ruminant excreta modifications depends on the C input quantity (Tables 6 and 7). The poaching effect could not be considered without taking into account the soil moisture saturation modification, as it showed a lower performance than the default model version (Tables 5 and 6).

**Table 7:** Root mean square error (RMSE) and mean difference of simulations and observations (BIAS) for the combined modifications (soil moisture up to saturation and poaching; ruminant excreta and plant residues; soil moisture saturation and plant residues) in Mg C ha^-1^ to the model and grassland intensive site and model efficiency (EF) and RMSE across sites.

Site	Performance test	Soil moisture saturation and Poaching effect	Ruminant excreta and plant residues	Soil moisture saturation and plant residues
Laqueuille	BIAS	-7.79	-16.56	-4.58
	RMSE	6.67	13.43	4.67
Oensingen	BIAS	-2.95	-6.58	-0.05
	RMSE	4.32	9.64	0.07
Easter Bush	BIAS	-1.44	-2.43	-0.10
	RMSE	1.64	2.77	0.11
Solohead	BIAS	-7.96	-6.88	-2.45
	RMSE	5.76	5.03	2.24
All sites	RMSE	5.96	8.66	3.12
	EF	0.94	0.87	0.98

### Sensitivity analysis

A sensitivity analysis of RothC_4 was performed to assess the robustness of the modifications (plant residues quality, ruminant excreta quality and soil moisture up to saturation) made in the different model versions presented. In general, RothC_4 seems to be more sensitive to C input quantity than to quality and to soil moisture function, particularly at saturation conditions.

The sensitivity analysis performed for resistant plant residues pool with the RothC_4 version showed a sensitivity index varying between 0.8% for the Easter Bush site and 2.6% for Oensingen and Solohead research farm (Table 8). Although the model was not very sensitive to the quality of plant residues, adding this modification enhanced the results depending on the quantity of plant residues (Table 8). In this context, according to other studies [57,87,94], specifying plant C input quality depending on residues partitioning instead of using the default RothC ratio for DPM and RPM should enable more reliable modelling of SOM dynamics. In order to ensure the sensitivity of the model to the plant C inputs in terms of quantity, we assessed its sensitivity to the R:S ratio based on our extensive literature review for temperate grassland species (S3 Table in S1 File). The sensitivity shown by the model to plant residues was higher than the sensitivity to the plant residues quality (S4 Table in S1 File).

**Table 8:** Sensitivity index of varying resistant plant residues fraction, lignin content corresponding to animal excreta quality and the rate modifying factor for moisture from its minimum to maximum values in RothC_4 for the different study sites.

	Plant residues quality (Resistant fraction)			Animal excreta quality (Lignin content)			Rate modifying factor for soil moisture		
Site	Output (min)	Output (max)	Sensitivity index	Output (min)	Output (max)	Sensitivity index	Output (min)	Output (max)	Sensitivity index
**Laqueuille**	118.6	120.4	1.5%	119.1	120.4	1.1%	104.6	120	12.8%
**Oensingen**	67.2	69	2.6%	68	69	1.4%	61.6	69.7	11.6%
**Easter Bush**	87.6	88.4	0.8%	87.3	88.7	1.6%	85.3	89.6	4.8%
**Solohead**	143.8	147.6	2.6%	143.6	148.1	3%	139.4	150.4	7.3%

In relation to the sensitivity of the RothC_4 version to the animal excreta quality, the values of sensitivity index obtained for the different experiments were in general low (between 1.1% and 3%) (Table 8). So, the use of average value for the different animal excreta fractions does not really impact the results, as we implemented in EOM modification. As for plant residues, the greatest value for the Solohead research farm could respond to the higher C inputs derived from animal excreta that received Solohead research farm as compared to the other sites. In order to focus on RothC_4 sensitivity to animal excreta quality with relation to its quantity, we assumed an annual C input derived from animal excreta of about 2.5 t C ha^-1^ distributed between March and June for the remaining sites that receive smaller amount of organic fertiliser. As animal excreta quality in the RothC model is connected to its quantity, the sensitivity index of animal excreta quality increased as its quantity increased (S4 Table in S1 File). In this context, according to [95], RothC displayed a moderate sensitivity to variations in animal excreta quality, more specifically the ratio between decomposable and resistant pools.

Sensitivity index regarding soil moisture modification was higher compared with the other modifications reaching, for example 12.8% in the Laqueuille site (Table 8). The variation in the sensitivity among the different study sites depend on their soil properties. Therefore, the modified model is sensitive to the rate modifying factor for soil moisture up to saturation under temperate moist climate conditions. In this context [96], concluded that reliable prediction of carbon turnover requires that the soil moisture together with the soil temperature reduction functions of the model need to be valid for the environmental conditions.

### Sources of uncertainty and research needs

Although RothC_3 and RothC_4 simulations performed well in simulating SOC changes for the selected sites, there were limitations related to the uncertainty of, both, model inputs and modifications, and to the limitation of the data used for validation.

Regarding model inputs, uncertainty was mainly related to the lack of detailed measured data of C inputs derived from plant and/or animal origin. In this study, we used the average of available measured values (details can be found in the section “Input data for the model and main assumptions”). However, measured C inputs is not always available, so its value could be supplied via linkage with another model, considering the grazing effect (case of plant residues). It is important to point out that previous studies running RothC in grassland ecosystems overestimated C inputs [33] and there is a lack of detailed information on how plant residues were estimated and/or assumptions regarding their conversion to C inputs [33]. In particular, the estimation of below-ground C inputs is another major source of uncertainty for SOC modelling [97]. Indeed, belowground C inputs depend on multiple factors, including site-specific agronomic practices and the response of plant genotypes to them, and direct measurements of belowground C inputs is a challenging issue [34]. For instance, if we estimate R:S ratio according to Eq (5) with the measured values in Oensingen site, we found close values of 1.9 and 1.5, respectively. However, for the Solohead research farm, the values were more different with a measured R:S ratio of 0.88 compared to an estimated value of 2.1. Moreover, the use of pedotransfer equations for initialising SOC pools, as an alternative for soil physical fractionation, may represent another source of uncertainty [98]. Indeed, although the reliability of pedotranfer equations, they could reveal some errors which are in the range of measurement error for SOC [79].

Regarding model modifications, a linear decline in the rate modifying factor for soil moisture was assumed under saturation conditions, like in the ECOSSE model, as there was not sufficient evidence to suggest a more refined relationship as indicated by [40]. However, the effect of soil moisture on SOC dynamics is complex and non-linear [99], interacting with temperature effect [100]. Improvements could be achieved by using a more refined function based on robust field experiments in order to better represent the different grassland sites. Furthermore, our estimations of animal excreta quality, based on literature review, are not conclusive and further refinements based on experiments could be made as, for example, to account for animal intake quality to predict its excreta quality. Regarding the poaching effect modification, based on the literature review we made, the number of long-term experiments under temperate moist region is limited. Moreover, due to the complexity of the soil damage (i.e., poaching) in which many factors could be involved (i.e., soil, animal, plant) [101], it is difficult to generalise our findings. The lack of usable, mechanistic simulation models of soil deformation under hooves and wheels is partly due to the lack of appropriate conceptual understanding and theory of the complex soil mechanical processes involved as well as the shortage of good and relevant experimental data [67].

Our equations and values suggested for the different modifications are representative for the conditions of moist temperate intensive grasslands and other site-specific equations, that are tailored to the objective study site, could be used.

In our study, simulations of the different modifications were compared to measured data of different study sites. However, field measurements also have deviations, which are source of uncertainty as they are used as the scale to evaluate model performance [102].

As future improvements, measurement of the different input data to the model (e.g., plant residues) would maximise the accuracy of estimations. However, this technique involves time, cost and labour [103]. As an alternative non-destructive method, combining the process-based model RothC with machine learning techniques can successfully help infer additional information from incomplete data sets [104]. For instance, the machine learning algorithms based on remote sensing data, such as the ArtificialNeural Network as a powerful empirical modelling, could improve the estimation of above-ground biomass with higher accuracy [105,106].

For future work, our modifications could be reproduced and/or refined to improve assessments of SOC changes in managed grasslands under temperate climatic conditions not only at a plot level but also at regional level since grassland systems continue to be understudied at broader scales [82,104].

## Conclusions

This study adapted the RothC model to managed grasslands under temperate moist conditions. The proposed modifications to the model considered the incorporation of distinction for plant residues components (i.e., above- and below-ground residues and rhizodeposition) in terms of quantity and quality and distinction for ruminant excreta quality, the extension of soil moisture up to saturation conditions and, finally, the introduction of the livestock damaging effect (i.e., poaching) on plant residues under water saturation conditions. The moisture response modification and the partition of C inputs derived from plant residues components improved the model predictability, but plant residues and ruminant excreta quality modifications improved the model predictability at a lesser extent. Finally, poaching simulation did not improve the model, since it results in complex and multi-factorial effects in these temperate grasslands. These modifications do not impair the performance of the model under temperate conditions. Indeed, they represent a broadening in the capability of the RothC model to simulate managed grassland under temperate moist conditions. It must be kept in mind that although there was good agreement between results from modified model and measured data from different studies, validating against more sites would greatly improve model confidence.

## Supporting information

S1 File (DOCX)
