# Supplementary material for: Estimating soil organic carbon changes in managed temperate moist grasslands with RothC
Source: PLoS One. 2021 Aug 20;16(8):e0256219. doi: 10.1371/journal.pone.0256219 (PMC8378727; doi:10.1371/journal.pone.0256219)
Supplement: S1 File — (DOCX) [file pone.0256219.s001.docx]

**Supporting information**

**S1 Appendix. Model modification**

The conversion from soil water content to soil moisture deficit (SMD_i_, mm) used in RothC referred to [1] is given by the following equation [1]:

${SMD}_{i}=\left( {WC}_{i}- {WC}_{fc} \right)\times10 \times depth$ (S1)

Where WC_fc_ is the soil water content at field capacity, WC_i_ is the soil water content above field capacity.


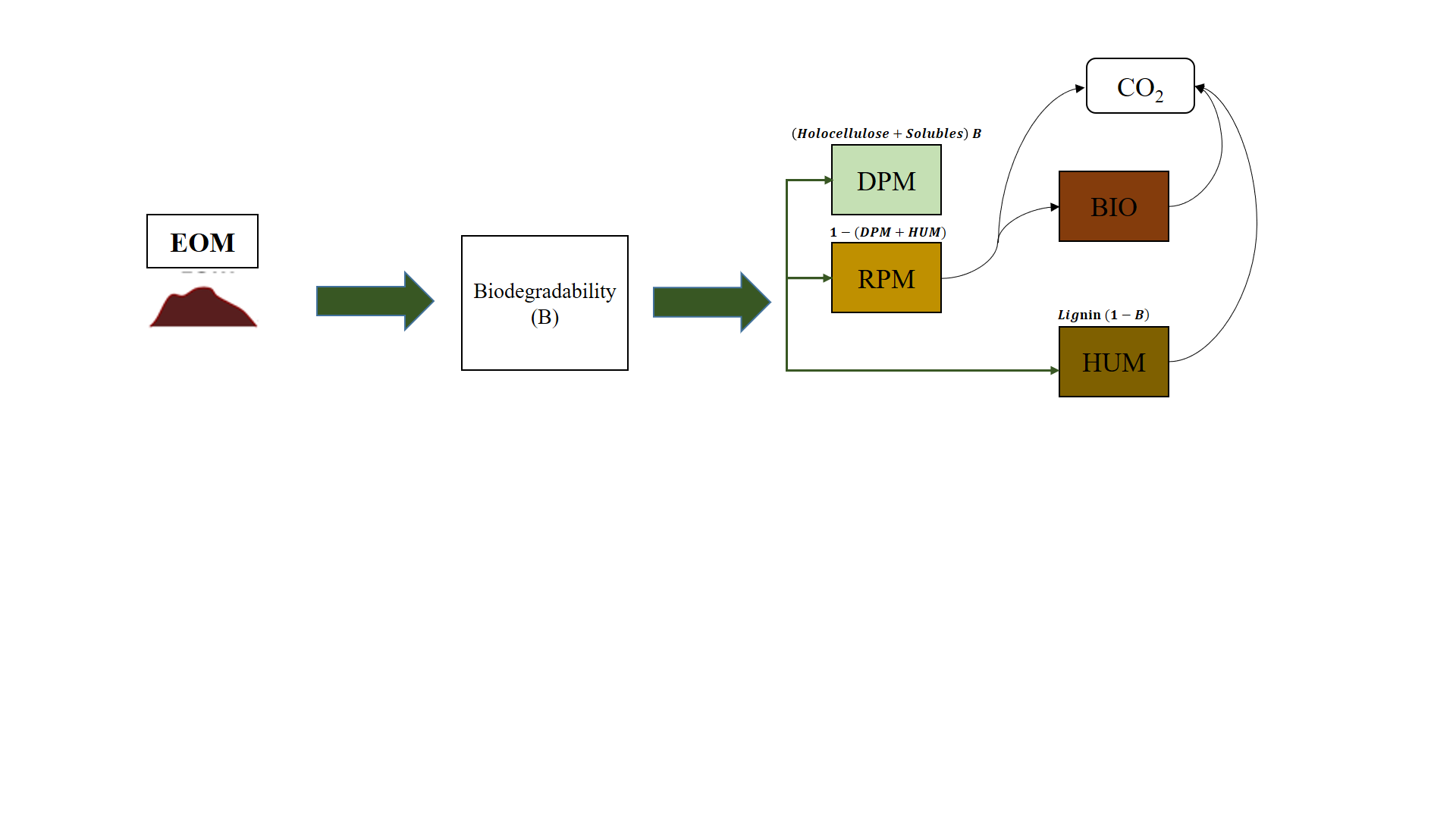


**S1 Fig. Structure of C input derived from EOM in RothC modified model.** (EOM, exogenous organic matter; DPM, decomposable EOM; RPM, resistant EOM; HUM, humified EOM)


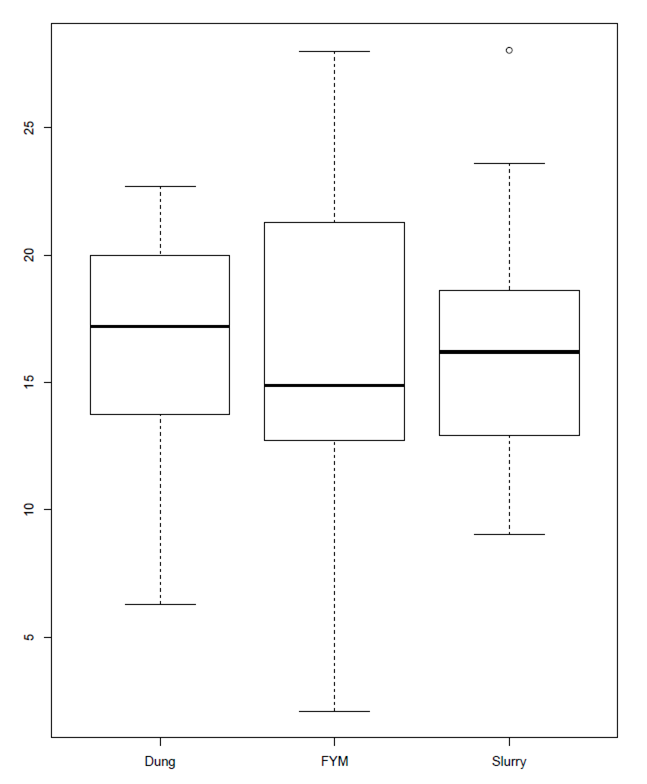


*FYM, Farmyard manure

**S2 Fig. Boxplot displaying the lignin Van Soest fraction variability for the different ruminant residue’s types (Dung, Farmyard manure, slurry), based on literature review findings**


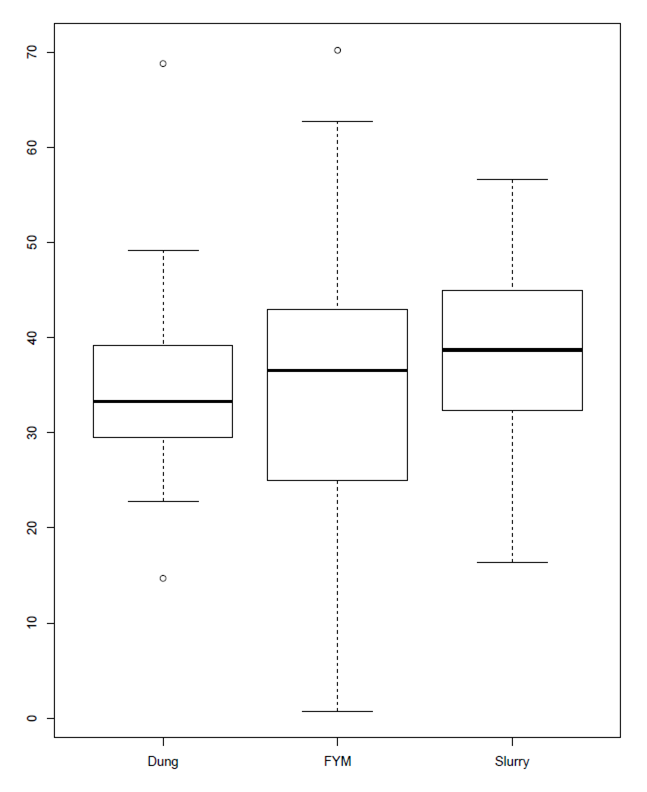


*FYM, Farmyard manure

**S3 Fig. Boxplot displaying soluble Van Soest fraction variability for the different ruminant residue types (Dung, Farmyard manure, slurry), based on literature review findings**


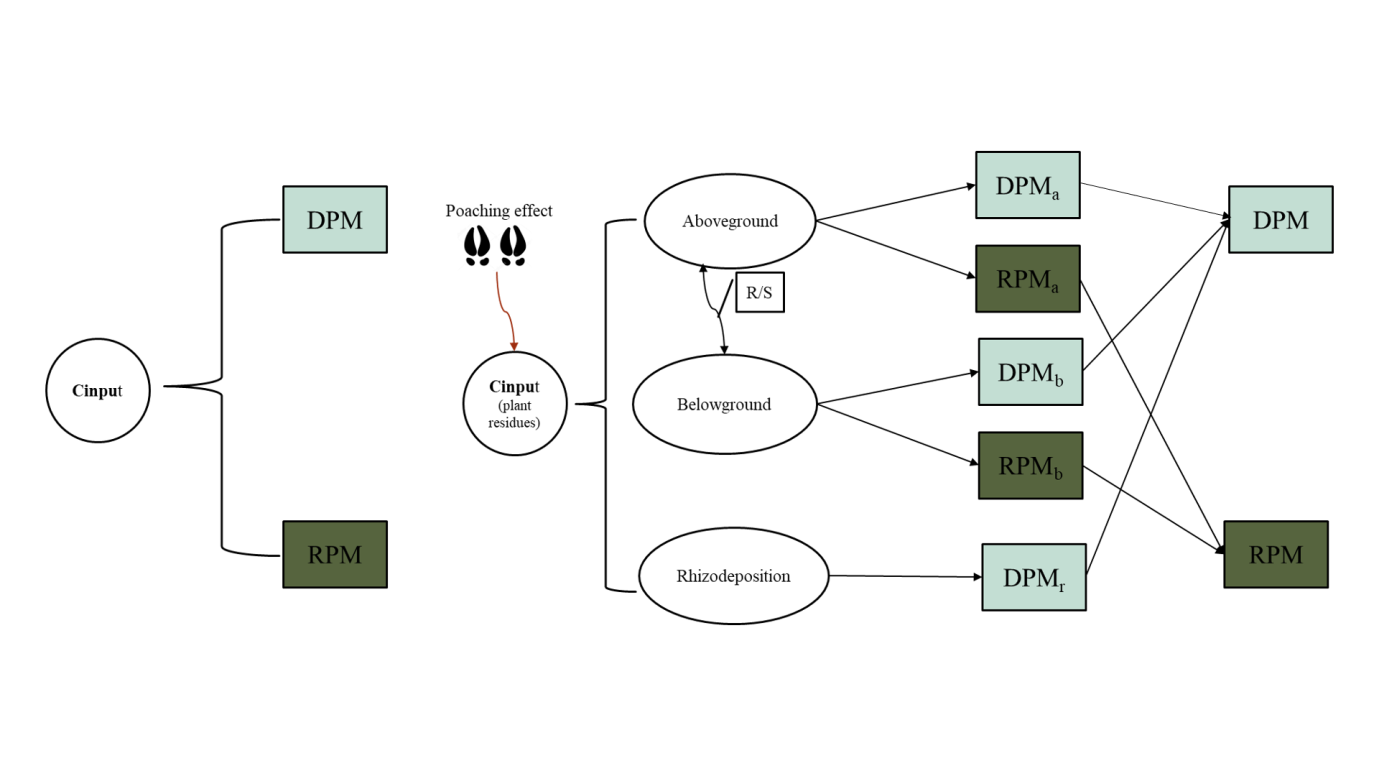


**S4 Fig. Structure of C input derived from plant residues in RothC modified model.** (DPM: decomposable plant material; RPM: resistant plant material; DPM_a_, decomposable above-ground plant material; RPM_a_, resistant above-ground plant material; DPM_b_, decomposable below-ground plant material; RPM_b_, resistant below-ground plant material; DPM_r_, decomposable rhizodeposits)

$Belowground Biomass=Aboveground Biomass \times R:S$ (S2)

$Belowground Residue =Belowground Biomass\times0.5$ (S3)

$Aboveground Residue =Aboveground Biomass\times fraction \left( Harvest or grazing \right)\times0.5$ (S4)

**Equations of cattle poaching effect modification**


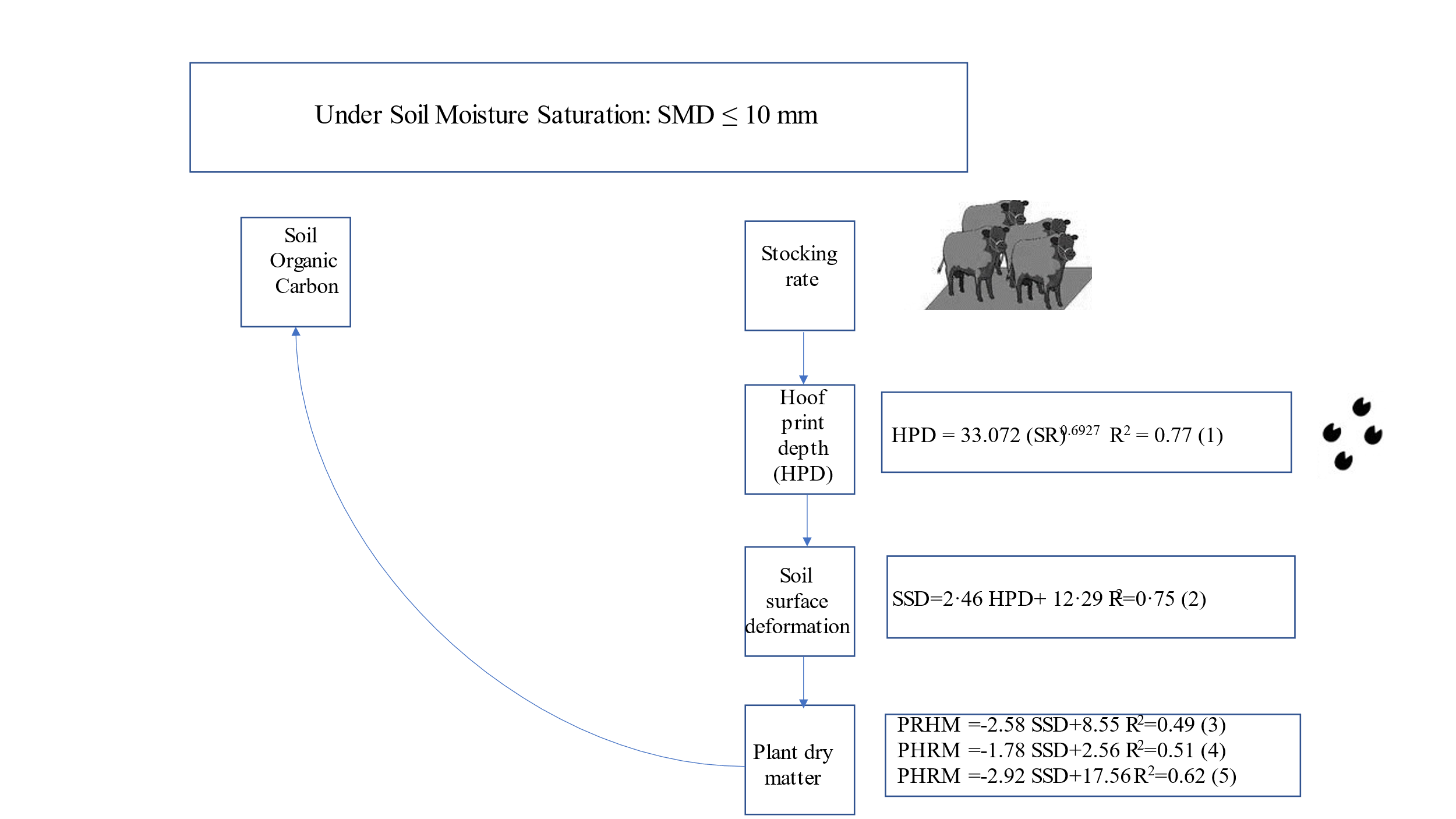


**S5 Fig. Conceptual diagram of how the animal trampling effect is simulated to affect SOC dynamics.**

**Hoof print depth** (HPD) is function of stocking rate (SR) depending on the soil texture.

HPD is expressed in mm and stocking rate (SR) is expressed in number of cows/ha (Average live weight =550kg). Equations are deduced from experiments in [2].

For example, for poorly drained soils: HPD = 33.072 (SR)^0.6927^ R^2^ = 0.77 (S5)

**Soil surface deformation** (SSD) is significantly correlated with HPD (SSD=2·46 HPD+ 12·29 R^2^=0·75)(A2) [3]. It is expressed in m/m.

**The proportional reduction in herbage dry mass** (PRHM) following each treading event. This proportion is without unit and was applied for plant C input according to [4] equations.

PRHM = -2.58 SSD+8.55 R^2^=0.49 (Early-spring turnout with an annual fertilizer input of 100 kg N ha^-1^). (S6)

PHRM = -1.78 SSD+2.56 R^2^=0.51(Early-spring turnout with no Fertilizer-Ninput). (S7)

PHRM = -2.92 SSD+17.56 R^2^=0.62 (Late-spring turnout with no Fertilizer-Ninput). (S8)

Where SSD is expressed in cm/m.

**S2 Appendix. Study sites description and input data**

1. *Study sites description*

The Laqueuille intensive site is a semi-natural grazing grassland (2.81 ha). The soil is classified as Andosol (20% clay, 53% silt and 27% sand) (FAO classification). The site was continuously grazed by heifers (1.1 SR/ha/yr) from May to October without additional feed supply, and fertilized with 210 kg N ha^-1^year^-1^ (ammonium nitrate) in three splits (more details on [5]and [6]). The Oensingen intensive site is cutting grassland. The soil is classified as Stagnic Cambisol (Eutric) (FAO, ISRIC and ISSS, 1998). The field has been sown with grass- clover mixtures since 2001 and is mown 4 times per year and fertilised with 214 kg N ha^-1^year^-1^ (as solid ammonium nitrate or liquid cattle manure) at the beginning of each growing cycle [7]. The Easter Bush experimental site is under permanent grassland grazing management. The soil is classified as Eutric Cambisol (FAO classification) and is imperfectly drained. Grazing in this site occurs all year round by heifers in calf, ewes and lamb, which always have access to the entire field (more details on [8] and [9]. The Solohead site is a dairy research farm with poorly drained soils. From 2004 to 2011, a typical grassland management involved rotational grazing [10].

1. *Input data for the model and main assumptions*

Average monthly temperature and precipitation for Laqueuille, Oensingen and Easter Bush sites were obtained from onsite Meteorological Stations for the periods 2004-2012, 2004-2011 and 2004-2010 respectively. For Solohead dairy farm, climatic data were provided by the Irish Meteorological Service referring to the nearest synoptic station with available climatic data for the simulation period 2004-2011. Monthly potential evapotranspiration was estimated using Thornthwaite equations [11] in case of non-availability of data.

**S3 Appendix. Modified model performance**

**S1 Table. Model performance measurement indices**

| **Performance measure** | **Equation** | **Unit** | **Value range and purpose** |
| --- | --- | --- | --- |
| **BIAS, mean difference of simulations and observations**  [12] | *BIAS =* $\bar{P}-\bar{O}$ | Unit of the variable | negative to positive infinity: the closer the values are to 0, the better the model (negative values: underestimation; positive values: overestimation) |
| **RMSE, Root Mean Square Error**  [12] | *RMSE =* $\frac{100}{\bar{O}} \times\sqrt{\frac{\sum_{i=1}^{n} {(Oi-Pi)}^{2}}{n}}$ | % | 0 to positive infinity: the closer the values are to 0, the better the model |
| **EF, Model efficiency**  [12] | *EF = 1 -* $\frac{\sum_{i=1}^{n} {(Pi-Oi)}^{2}}{\sum_{i=1}^{n} {(Oi-\bar{O)}}^{2}}$ | - | Negative infinity to 1 (optimum): the closer the  values are to 1, the better the model |

P, predicted value; O, measured value; n, number of P/O pairs; i, each of P/O pairs; O, mean of measured values; P, mean of predicted values.

**S4 Appendix. Sensitivity analysis**

**S2 Table. NDF range of perennial ryegrass.**

| **NDF min** | **NDF max** | **Reference** |
| --- | --- | --- |
| 48.9 | 52.2 | [13] |
| 36.5 | 53.5 | [14] |
| 40.7 | 49.4 | [15] |
| 45.8 | 58.5 | [16] |
| 43 | 52.7 | [17] |
| 47.8 | 57.4 | [18] |
| 41.1 | 54 | [19] |
| 44 | 63.2 | [20] |
| 39 | 53 | [21] |
| 41.4 | 69.3 | [22] |
| 38.7 | 42.5 | [23] |
| 39.4 | 57.8 | [24] |
| 46.1 | 55.2 | [25] |
| 32 | 47 | [26] |
| 49.8 | 57.4 | [27] |

**S3 Table.** **Sensitivity index of varying lignin content corresponding to animal excreta quality from its minimum to maximum values in RothC_4 for the different study sites under C input quantity (derived from animal excreta) scenario of 2.5 t C ha^-1^ year^-1^**

| **Site** | **Output**  **(min value)** | **Output**  **(max value)** | **Sensitivity index**  **(%)** |
| --- | --- | --- | --- |
| **Laqueuille**  **(2.5 t C ha^-1^)** | 127.0 | 133.1 | 4.7 |
| **Oensingen**  **(2.5 t Cha^-1^)** | 74.2 | 79.5 | 6.6 |
| **Easter Bush**  **(2.5 t C ha^-1^)** | 91.7 | 96.8 | 5.3 |

1. Farina R, Coleman K, Whitmore AP. Modification of the RothC model for simulations of soil organic C dynamics in dryland regions. Geoderma. 2013;200–201: 18–30. doi:10.1016/j.geoderma.2013.01.021

2. Tuñon G, O’Donovan M, Lopez Villalobos N, Hennessy D, Kemp P, Kennedy E. Spring and autumn animal treading effects on pre-grazing herbage mass and tiller density on two contrasting pasture types in Ireland. Grass Forage Sci. 2014;69: 502–513. doi:10.1111/gfs.12055

3. Tuohy P, Fenton O, Holden NM, Humphreys J. The effects of treading by two breeds of dairy cow with different live weights on soil physical properties, poaching damage and herbage production on a poorly drained clay-loam soil. J Agric Sci. 2014;153: 1424–1436. doi:10.1017/S0021859614001099

4. Phelan P, Keogh B, Casey IA, Necpalova M, Humphreys J. The effects of treading by dairy cows on soil properties and herbage production for three white clover-based grazing systems on a clay loam soil. Grass Forage Sci. 2013;68: 548–563. doi:10.1111/gfs.12014

5. Klumpp K, Tallec T, Guix N, Soussana JF. Long-term impacts of agricultural practices and climatic variability on carbon storage in a permanent pasture. Glob Chang Biol. 2011;17: 3534–3545. doi:10.1111/j.1365-2486.2011.02490.x

6. Touhami H Ben, Lardy R, Barra V, Bellocchi G. Screening parameters in the Pasture Simulation model using the Morris method. Ecol Modell. 2013;266: 42–57. doi:10.1016/j.ecolmodel.2013.07.005

7. Ammann C, Spirig C, Leifeld J, Neftel A. Assessment of the nitrogen and carbon budget of two managed temperate grassland fields. Agric Ecosyst Environ. 2009;133: 150–162. doi:10.1016/j.agee.2009.05.006

8. Skiba U, Jones SK, Drewer J, Helfter C, Anderson M, Dinsmore K, et al. Comparison of soil greenhouse gas fluxes from extensive and intensive grazing in a temperate maritime climate. Biogeosciences. 2013;10: 1231–1241. doi:10.5194/bg-10-1231-2013

9. Jones S, Helfter C, Anderson M, Coyle M, Campbell C, Famulari D, et al. The nitrogen, carbon and greenhouse gas budget of a grazed, cut and fertilised temperate grassland. Biogeosciences Discuss. 2016; 1–55. doi:10.5194/bg-2016-221

10. Necpálová M, Li D, Lanigan G, Casey IA, Burchill W, Humphreys J. Changes in soil organic carbon in a clay loam soil following ploughing and reseeding of permanent grassland under temperate moist climatic conditions. Grass Forage Sci. 2013;69: 611–624. doi:10.1111/gfs.12080

11. THORNTHWAITE CW. An Approach Toward a Rational. Geogr Rev. 1948;38: 55–94.

12. Smith JU, Smith P. Environmental Modelling. An Introduction. Oxford Uni. Oxford; 2007.

13. Boudon A, Peyraud J. The release of intracellular constituents from fresh ryegrass ( Lolium perenne L .) during ingestive mastication in dairy cows : effect of intracellular constituent , season and stage of maturity. Anim Feed Sci Technol. 2001;93: 229–245.

14. De Boever JL, Dupon E, Wambacq E, Latré J. The effect of a mixture of Lactobacillus strains on silage quality and nutritive value of grass harvested at four growth stages and ensiled for two periods. Agric Food Sci. 2013;22: 115–126. doi:10.23986/afsci.6709

15. Elgersma A, Søegaard K. Effects of species diversity on seasonal variation in herbage yield and nutritive value of seven binary grass-legume mixtures and pure grass under cutting. Eur J Agron. 2016;78: 73–83. doi:10.1016/j.eja.2016.04.011

16. Ergon A, Kirwan L, Fystro G, Bleken MA, Collins RP, Rognli OA. Grass and Forage Science Species interactions in a grassland mixture under low nitrogen fertilization and two cutting frequencies . II . Nutritional quality. Grass Forage Sci. 2016; 1–10. doi:10.1111/gfs.12257

17. Frandsen KJ. Variability and Inheritance of Digestibility in Perennial Ryegrass ( Lolium perenne ), Meadow Fescue ( Festuca pratensis ) and Cocksfoot ( Dactylis glomerata ). Acta Agric Scand. 1986;36: 241–263. doi:10.1080/00015128609436528

18. Küchenmeister K, Küchenmeister F, Kayser M, Wrage-Mönnig N, Isselstein J. Influence of drought stress on nutritive value of perennial forage legumes. Intrnational J Plant Prod. 2013;7: 1735–8043.

19. Lee MRF, JONES EL, Jonathan M. MOORBY, Mervyn O. HUMPHREYS MKT, MACRAE JC, SCOLLAN ND. Original article Production responses from lambs grazed on Lolium perenne selected for an elevated water-soluble carbohydrate concentration. Anim Res. 2002;50: 441–449.

20. Ohlsson C, Houmøller LP, Weisbjerg MR, Lund P, Hvelplund T. Effective rumen degradation of dry matter , crude protein and neutral detergent fibre in forage determined by near infrared reflectance spectroscopy. J Anim Physiol Anim Nutr (Berl). 2007;91: 498–507. doi:10.1111/j.1439-0396.2007.00683.x

21. Purcell PJ, Brien MO, Boland TM, Mcevoy M, Grogan D, Kiely PO. Grass and Forage Science In vitro rumen methane output of perennial ryegrass varieties and perennial grass species harvested throughout the growing season. Grass Forage Sci. 2012; 1–19. doi:10.1111/j.1365-2494.2011.00845.x

22. Salama H, Loesche M, Herrmann A, Taube F, Gierus M. A simplified maturity index to quantify the development stage of perennial ryegrass ( Lolium perenne L .) and its relationship with yield and nutritive value. J L Manag Food Environ. 2017;68: 89–101. doi:10.1515/boku-2017-0009

23. Van Vuuren AM, Van Der Koelen CJ, Vroons-De Bruin J. Ryegrass Versus Corn Starch or Beet Pulp Fiber Diet Effects on Digestion and Intestinal Amino Acids in Dairy Cows. J Dairy Sci. 1993;76: 2692–2700. doi:10.3168/jds.S0022-0302(93)77605-5

24. Armstrong RH, Common TG, Smith HK. The voluntary intake and in vivo digestibility of herbage harvested from indigenous hill plant communities. Grass Forage Sci. 1986;41: 53–60. doi:10.1111/j.1365-2494.1986.tb01792.x

25. Østrem L, Volden B, Steinshamn H, Volden H. Festulolium fibre characteristics and digestibility as affected by maturity. Grass Forage Sci. 2014;70: 341–352. doi:10.1111/gfs.12126

26. Salama H, Lösche M, Herrmann A, Gierus M, Loges R, Feuerstein U, et al. Limited genotype- and ploidy-related variation in the nutritive value of perennial ryegrass (Lolium perenne L.). Acta Agric Scand Sect B Soil Plant Sci. 2012;62: 23–34. doi:10.1080/09064710.2011.563750

27. Sun XZ, Waghorn GC, Clark H. Cultivar and age of regrowth effects on physical, chemical and in sacco degradation kinetics of vegetative perennial ryegrass (Lolium perenne L.). Anim Feed Sci Technol. 2010;155: 172–185. doi:10.1016/j.anifeedsci.2009.12.004
